# Salvianolic acid B inhibits myocardial I/R-induced ROS generation and cell apoptosis by regulating the TRIM8/GPX1 pathway

**DOI:** 10.1080/13880209.2022.2096644

**Published:** 2022-08-14

**Authors:** Bo Lu, Jianhua Li, MingTai Gui, Lei Yao, Mingsong Fan, Xunjie Zhou, Deyu Fu

**Affiliations:** a Department of Cardiology, Yueyang Hospital of Integrated Traditional Chinese and Western Medicine, Shanghai University of Traditional Chinese Medicine, Shanghai, China; b Shanghai Leiyunshang Pharmaceutical Co., Ltd., Shanghai, China

**Keywords:** E3 ligase, myocardial ischemia/reperfusion, ubiquitination

## Abstract

**Context:**

Salvianolic acid B (SalB) can attenuate myocardial ischemia/reperfusion (I/R) injury, but the mechanisms are not entirely known.

**Objective:**

Our study investigates if SalB protects cardiomyocytes against I/R injury by regulating Tripartite motif (TRIM) protein.

**Materials and methods:**

AC16 cardiomyocytes were treated with I/R, and then with SalB (10, 25 and 50 μM) for 24 h, while control cells were cultured under normal conditions. Female Sprague-Dawley rats were subjected to I/R injury, and then intravenously injected with 20, 40, or 60 mg/kg SalB or saline, as a control, rats received sham operation and saline injection.

**Results:**

Upon treatment, apoptotic rate, reactive oxygen species (ROS), and malondialdehyde (MDA) were increased 10-, 3.8-, and 1.3-fold, respectively, while superoxide dismutase (SOD) activity was reduced by 62.1% compared to control cells. I/R treatment elevated the mRNA and protein expression of TRIM8. SalB treatment remarkably abolished the above-mentioned effects of I/R treatment. TRIM8 knock-down could partially alleviate I/R-induced myocardial injury. TRIM8 overexpression promoted cardiomyocyte injury, which was alleviated by SalB. Moreover, TRIM8 negatively regulated protein expression of antioxidant enzyme glutathione peroxidase 1 (GPX1). TRIM8 protein interacted with GPX1 and TRIM8 overexpression promoted GPX1 ubiquitnation. GPX1 knock-down abolished the protective effects of SalB on I/R-injured cardiomyocytes. Our *in vivo* experiments confirmed the effects of SalB on I/R-induced myocardial injury.

**Discussion and conclusions:**

SalB protected cardiomyocytes from I/R-induced apoptosis and oxidative stress *in vitro* and *in vivo*, which was partly mediated by the TRIM8/GPX1 axis. This suggests that down-regulation of TRIM8 expression may ameliorate I/R-induced myocardial injury.

## Introduction

Myocardial infarction, which occurs when the cardiac muscle doesn’t get enough oxygen due to decreased blood flow, can cause severe heart damage (Thackeray et al. 2018). Early reperfusion is an effective therapy to restore the supply of oxygen in the ischaemic area. However, the reperfusion itself can cause secondary damage to the heart, which has been known as myocardial ischemia/reperfusion (I/R) injury (DeBerge et al. 2017). The pathological mechanisms of myocardial I/R injury are complex, and many biological processes are involved, including oxidative stress, inflammatory reaction, and cardiomyocyte apoptosis (Al-Salam and Hashmi 2018). Recently, many compounds extracted from medicinal herbs have been found to alleviate the myocardial I/R injury, such as saponins and rosmarinic acid (Han et al. 2017; He et al. 2018).

Salvianolic acid B (SalB) (Figure S1), which is extracted from *Salvia miltiorrhiza* Bunge (Lamiaceae) (Danshen in Chinese), is a bioactive phenolic compound that has antioxidant and free radical scavenging properties (Dong et al. 2010). It has been found to exert protective effects in many diseases. For example, SalB is a tumour suppressor which can inhibit cancer cell growth and promote its apoptosis (Zhao et al. 2010; Jing et al. 2016). In neurodegenerative diseases, SalB exerts a neuroprotective role by inhibiting neuroinflammation and neuronal apoptosis (Zhao et al. 2019). Existing studies have observed that SalB can attenuate myocardial I/R injury (Qiao and Xu 2016; Liu et al. 2019). However, the detailed regulatory mechanisms are not entirely known.

Tripartite motif (TRIM) proteins are a class of E3 ubiquitin ligases that can mediate ubiquitin-dependent protein degradation, thereby being involved in many cellular processes (Hatakeyama 2017). For instance, TRIM11 can promote proliferation and repress apoptosis in colon cancer cells (Yin et al. 2016). TRIM32 can induce reactive oxygen species (ROS) generation and neuronal apoptosis in an *in vitro* cerebral I/R model (Wei et al. 2019). Recent studies have revealed that TRIM proteins are important regulators in the pathological process of heart diseases. For example, TRIM8 is remarkably up-regulated in I/R-injured cardiomyocytes, and can induce ROS generation and cell apoptosis (Dang et al. 2020). TRIM33 can enhance oxidative stress via mediating the ubiquitination of antioxidant enzyme glutathione peroxidase 1 (GPX1) during myocardial I/R injury (Jian et al. 2016).

Our study explores whether SalB protects cardiomyocytes against I/R injury by regulating TRIM proteins. An *in vitro* myocardial I/R model was established by culturing AC16 cardiomyocytes with hypoxia/reoxygenation treatment. We evaluated the effects of SalB on I/R-induced oxidative stress and apoptosis in AC16 cells through TRIM8/GPX1 axis. Further *in vivo* experiments on the rat model of myocardial I/R injury were also done.

## Materials and methods

### Chemicals

SalB (purity ≥ 98%; Cat. No. S101148) was obtained from Aladdin Company (Shanghai, China).

### Cell culture and treatment

Human cardiomyocyte cell line AC16 was purchased from ATCC (Rockville, MD, USA). The cardiomyocytes were cultured in Dulbecco's Modified Eagle's Medium (DMEM) (Sigma, France) with 10% foetal bovine serum (Gibco; Carlsbad, CA, USA) at 5% CO_2_ at 37**°**C. For I/R treatment, AC16 cells were cultured in serum/glucose-free DMEM under hypoxia (94% N_2_, 1% O_2,_ and 5% CO_2_) for 5 h, followed by culture in normal medium under reoxygenation (5% CO_2_ and 95% O_2_) for 1 h (Zhang et al. 2018). Cells that were cultured under normal conditions were used as control. For SalB administration, AC16 cells were cultured with different concentrations of SalB (10, 25 and 50 μM) for 24 h.

### Overexpression and knock-down of TRIM8 and GPX1

Lentivirus vector overexpressingTRIM8 was commercially constructed (Genewiz, Suzhou, China). Lentivirus was generated as previously described (Zhang et al. 2021). In brief, 293 T cells were transfected with TRIM8 gene-inserted vector plasmid pLVX-Puro, and two packaging plasmids psPAX2 and pMD2G. Forty-eight h later, high-titer recombinant lentiviruses were obtained. To overexpress TRIM8 in AC16 cells, the cells were cultured in DMEM containing lentivirus oeTRIM8 at a multiplicity of infection of 10 for 12 h, and then cultured in fresh medium for another 12 h. The short hairpin RNAs (shRNAs) of TRIM8 and GPX1 were obtained from Hanbio (Shanghai, China), and their sequences were listed in Table 1. To knock down TRIM8 or GPX1 in AC16 cells, the cells were transfected with corresponding shRNA using Lipofectamine 2000 (Invitrogen; Grand Island, NY, USA).

**Table 1.** The sequences of TRIM8 and GPX1 shRNAs.

shRNA	Sense (5′-3′)	Antisense (5′-3′)
shTRIM8-1	CCAACAUCGUGGAGAAGUUTT	AACUUCUCCACGAUGUUGGTT
shTRIM8-2	GGAUUUCUACAGGGUGUAUTT	AUACACCCUGUAGAAAUCCTT
siNC-TRIM8	CAGUACUUUUGUGUAGUACAA	UUGUACUACACAAAAGUACUG
shGPX1-1	GCAAGGUACUACUUAUCGATT	UCGAUAAGUAGUACCUUGCTT
shGPX1-2	GCUUCCAGACCAUUGACAUTT	AUGUCAAUGGUCUGGAAGCTT
shGPX1-3	GGUGUUUCCUCUAAACCUATT	UAGGUUUAGAGGAAACACCTT
siNC-GPX1	CAGUACUUUUGUGUAGUACAA	UUGUACUACACAAAAGUACUG

### Flow cytometric analyses

Apoptosis and ROS levels were evaluated with flow cytometric analyses as previously described (Zhang et al. 2021). AC16 cells were digested with 0.25% trypsin-EDTA (Beyotime, China). After centrifugation at 1000 rpm for 5 min, the cells were collected and re-suspended in PBS. For the assessment of apoptosis, 5 × 10^5^ cells were treated with 20 μL Annexin V-FITC (BD Pharmingen; San Diego, CA, USA) for 20 min, followed by incubation with 10 μL propidium iodide for 10 min. Finally, apoptotic cells were detected using a flow cytometer (Becton Dickinson; Franklin Lakes, NJ, USA) as previously described (Zhang et al. 2021). For the measurement of ROS level, 5 × 10^5^ cells were incubated with 5 µM 2′,7′-dichlorodihydrofluorescein diacetate (DCFH-DA) probe (Calbiochem; San Diego, CA, USA) for 30 min at 37 °C. The fluorescence intensity was detected using a flow cytometer (excitation: 480 nm; emission: 525 nm).

### Detection of superoxide dismutase (SOD) activity, and malondialdehyde (MDA) level

SOD activity and MDA level in AC16 cells were detected using xanthine oxidase and thiobarbituric acid methods, respectively (Cao et al. 2018). Both of these experiments were performed with commercial kits from Hanbio (Shanghai, China) according to the instructions of the manufacturer.

### Quantitative real-time PCR (RT-qPCR)

Total RNA was collected from AC16 cells and reverse-transcribed into cDNA with the 1st Strand cDNA Synthesis Kit (TaKaRa, Japan). RT-qPCR was performed with SYBR Green kit (Qiagen, Germany) as per the following workflow: 95 °C for 10 min; 40 cycles of 95 °C for 15 s and 60 °C for 45 s. The mRNA level was normalised to GAPDH using the ^ΔΔ^CT method (Zhang et al. 2018). The used primers were shown in Table 2.

**Table 2.** The used primers in RT-qPCR.

Gene	Forward primers (5′-3′)	Reverse primers (5′-3′)
TRIM8	CAGCCGTCCACCAAACACTAC	ACCTCTGCGTCCAGGAGATTC
TRIM11	CACCTAAGCTGCACAGTTCC	GGCTGCCTCCTAATTCTTCC
TRIM32	TAACTCGTCTGCGGGAAC	CTCTGCTCCTCTACCACTTG
TRIM33	TACAGCAAGCGACTGATTAC	TGCCCAACTACAACATTAGG
GPX1	AGTCGGTGTATGCCTTCTC	CTTCGTTCTTGGCGTTCTC
GAPDH	AATCCCATCACCATCTTC	AGGCTGTTGTCATACTTC

### Western blotting

Total protein was collected from AC16 cells and quantified using a bicinchoninic acid (BCA) protein assay kit (Gibco, USA). Protein (2.5 μg) was separated by sodium dodecyl sulphate polyacrylamide gel electrophoresis (SDS-PAGE) and transferred onto a polyvinylidene fluoride (PVDF) membrane (Solvay Pharmaceuticals; Marietta, GA, USA). After incubation with 5% non-fat milk at 4 °C overnight, the membranes were incubated overnight at 4 °C with antibodies against TRIM8 (1:1000; Abcam; Cambridge, MA, USA), TRIM11 (1:2000; Abcam), TRIM32 (1:1000; Abcam), TRIM33 (1:1000; Cell Signalling Technology; Danvers, MA, USA), GPX1 (1:200; Abcam), and GAPDH (1:2000; Cell Signalling Technology). Then they were incubated with HRP-conjugated secondary antibody (1:1000; Abcam) for 1 h at 37 °C. Finally, the target proteins were visualised with enhanced chemiluminescent substrates (Beyotime) as previously described (Zhang et al. 2018).

### Co-Immunoprecipitation and ubiquitination assays

In order to detect interaction between TRIM8 and GPX1, co-immunoprecipitation assay was performed (Zhang et al. 2021). Equal amount of total protein was incubated with IgG, TRIM8, and GPX1 overnight at 4 °C, and then were incubated with Protein A/G Plus-Agarose beads for 2 h at 4 °C (Millipore; Bredford, MA, USA). Thereafter, the beads were washed by PBS and then boiled in loading buffer for 5 min. The supernatant was collected for subsequent immunoblotting. To assess the ubiquitination level of GPX1, ubiquitination assay was performed. The workflow of ubiquitination assay was similar with co-immunoprecipitation assay. Briefly, anti-GPX1 antibody was used to pull down immunocomplex in AC16 cells, and subsequent western blotting was performed with anti-ubiquitin antibody (1:1000; CST) as primary antibody.

### Animal experiments

All animal experiments were performed in accordance with ARRIVE guidelines for the use of laboratory animals. A total of 50 female Sprague-Dawley rats (300 ± 25 g) were purchased from Kay Biological Technology Co. LTD (Shanghai, China). All rats were held under constant temperature (20 ± 1 °C) and humidity (40-50%) with 12 h artificial light daily and free access to food and water. One week later, the rats were randomly divided into five groups: (1) Control: rats received sham operation and saline injection; (2) I/R: rats received I/R treatment and saline injection; (3) I/R + SalB_L: rats received I/R treatment and injection of 20 mg/kg SalB; (4) I/R + SalB_M: rats received I/R treatment and injection of 40 mg/kg SalB; (5) I/R + SalB_H: rats received I/R treatment and injection of 60 mg/kg SalB. I/R treatment was performed following the workflow described by Tang et al. (2017). In brief, rat’s coronary artery was clamped with a plastic tube for 60 min, and then the coronary artery was restored by releasing the clamp. Three hours later, I/R-treated rats were injected intravenously with SalB or saline. Twenty-seven hours later, rats were sacrificed and cardiac tissue was collected. The animal experiments were approved by Yueyang Hospital of Integrated Traditional Chinese and Western Medicine, Shanghai University of Traditional Chinese Medicine (No. YYLAC-2021-119).

### TUNEL [terminal deoxynucleotidyltransferase (TdT)-mediated dUTP nick end-labeling] assay

Cardiac tissue was fixed in 10% formalin for 48 h, and then dehydrated in graded ethanol (50, 70, 85, 95 and 100%). After being embedded in paraffin, the tissue sample was cut into 5 μm sections. To detect apoptotic cells, TUNEL assay was performed (Zhang et al. 2021). Briefly, the paraffin section was incubated with 50 μl TUNEL reaction mixture for 1 h at 37**°**C, followed by incubation with 50 μl POD for 30 min at 37**°**C. Afterwards, the section was stained with 3,3′-diaminobenzidine (DAB) and counterstained with haematoxylin. The numbers of TUNEL-positive cells in five random fields were counted using a microscope (ECLIPSE Ni, Japan).

### Dihydroethidium staining

Cardiac tissue was fixed in 4% paraformaldehyde for 24 h, and then dehydrated with 15% and 30% sucrose solution. After being embedded with optimal cutting temperature (OCT) compound in liquid nitrogen, the tissue sample was cut into 8 μm frozen sections. Dihydroethidium (DHE) staining was applied to detect the ROS level(Lyu et al. 2021). The sections were stained with 10 μM DHE for 30 min at 37 °C, and then stained with 4′,6-diamidino-2-phenylindole (DAPI) for 5 min. Fluorescence intensity was assessed using a fluorescence microscopy (Olympus, Japan).

### Statistics

Experimental data of three independent repeats were shown as mean ± standard deviation (SD). Differences were analysed using one-way Analysis of variance (ANOVA) and Tukey test in GraphPad Prism version 7.0 (GraphPad; San Diego, CA, USA). *p*-values less than 0.05 have significance in statistics.

## Results

### SalB repressed I/R-induced apoptosis and oxidative stress in AC16 cells

**Figure 1.**
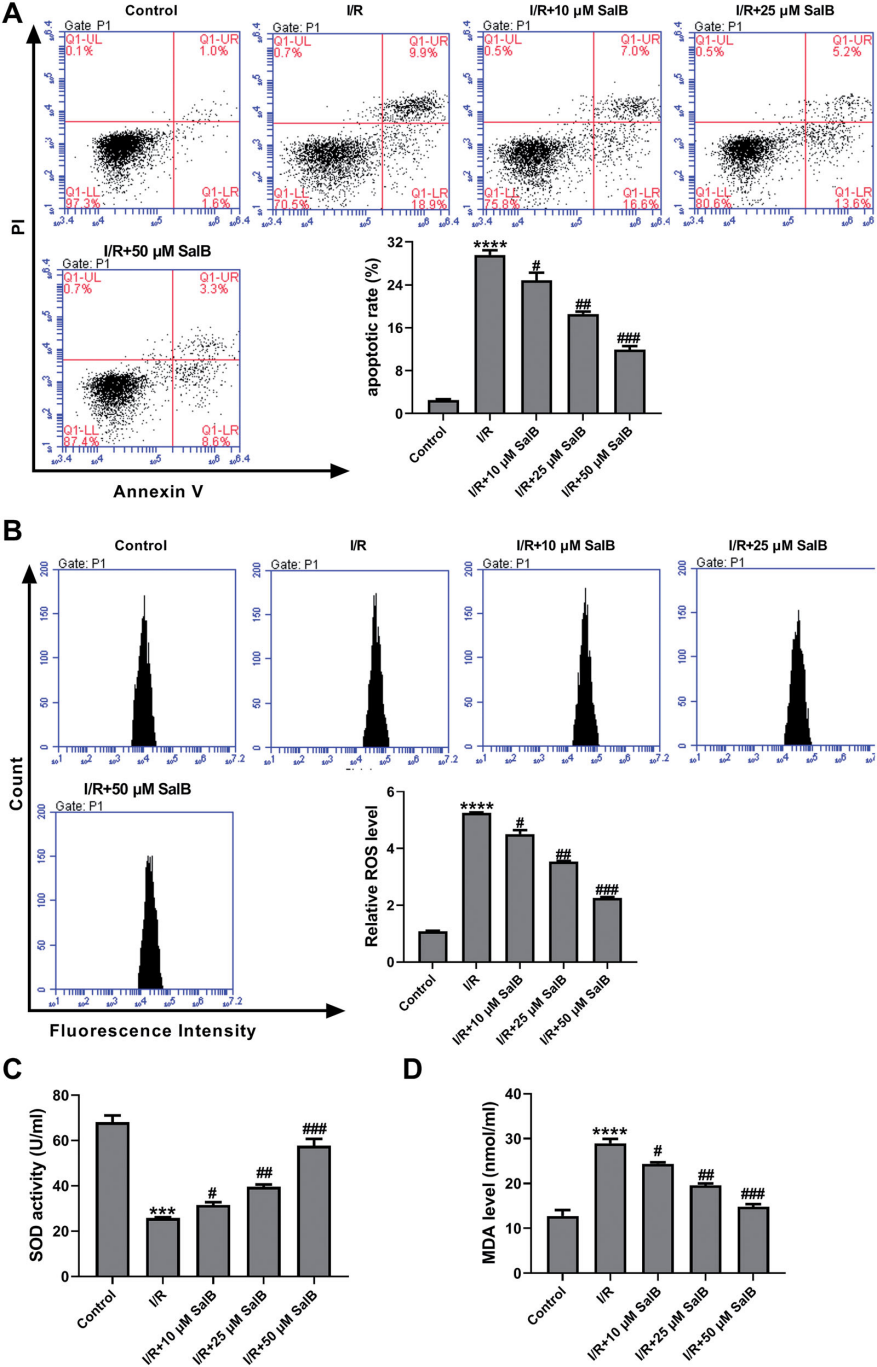
SalB repressed ischemia/reperfusion (I/R)-induced apoptosis and oxidative stress in AC16 cells. I/R-injured AC16 cells were treated with different concentrations of SalB. (A) Apoptotic detection was performed using a flow cytometry. (B) ROS level was detected with DCFH-DA probe. (C) SOD activity was detected using xanthine oxidase method. (D) MDA level was detected using TBA methods. ***p < 0.001 and ****p < 0.0001 vs. Control; #*p* < 0.05, ^##^*p* < 0.01, and ^###^*p* < 0.001 vs. I/R.

I/R-injured AC16 cells were treated with different doses of SalB, and cells that were cultured under normal conditions were used as control. Apoptotic cells and ROS level were detected using a flow cytometry. Results showed that apoptotic rate in cells with I/R treatment was increased 10-fold over control (I/R, 29.6% ± 0.9%; control, 2.5% ± 0.2%), while subsequent SalB administration to I/R-induced cardiomyocytes attenuated apoptosis (I/R + 10 µM SalB, 24.9% ± 1.4%; I/R + 25 µM SalB, 18.6% ± 0.5%; I/R + 50 µM SalB, 12.6% ± 0.7%) (Figure 1A). ROS level in AC16 cells with I/R treatment was increased to 5.2-fold of control cells, and dropped to 4.5-fold, 3.5-fold, and 2.3-fold of control cells when SalB was applied at 10 µM, 25 µM, and 50 µM, respectively (Figure 1B). SOD activity and MDA level in AC16 cells were also measured. Results showed that I/R treatment in AC16 cells reduced SOD activity by 62.1% (I/R, 25.78 ± 0.33 U/mL vs. Control, 68.01 ± 3.05 U/mL) and increased MDA level by 1.3-fold compared to control cells (I/R, 28.96 ± 1.00 nmol/L vs. Control, 12.76 ± 1.34 nmol/L), while SalB administration alleviated the effects of I/R treatment on SOD activity (I/R + 10 µM SalB, 31.51 ± 1.29 U/mL; I/R + 25 µM SalB, 39.58 ± 0.96 U/mL; I/R + 50 µM SalB, 57.62 ± 3.08 U/mL) and MDA level (I/R + 10 µM SalB, 24.39 ± 0.34 nmol/L; I/R + 25µM SalB, 19.58 ± 0.43 nmol/L; I/R + 50 µM SalB, 14.82 ± 0.60 nmol/L) (Figure 1C–D). Taken together, our data suggest that SalB inhibits I/R-induced apoptosis and oxidative stress in AC16 cells.

### SalB inhibited I/R-induced up-regulation of TRIM8 expression

**Figure 2.**
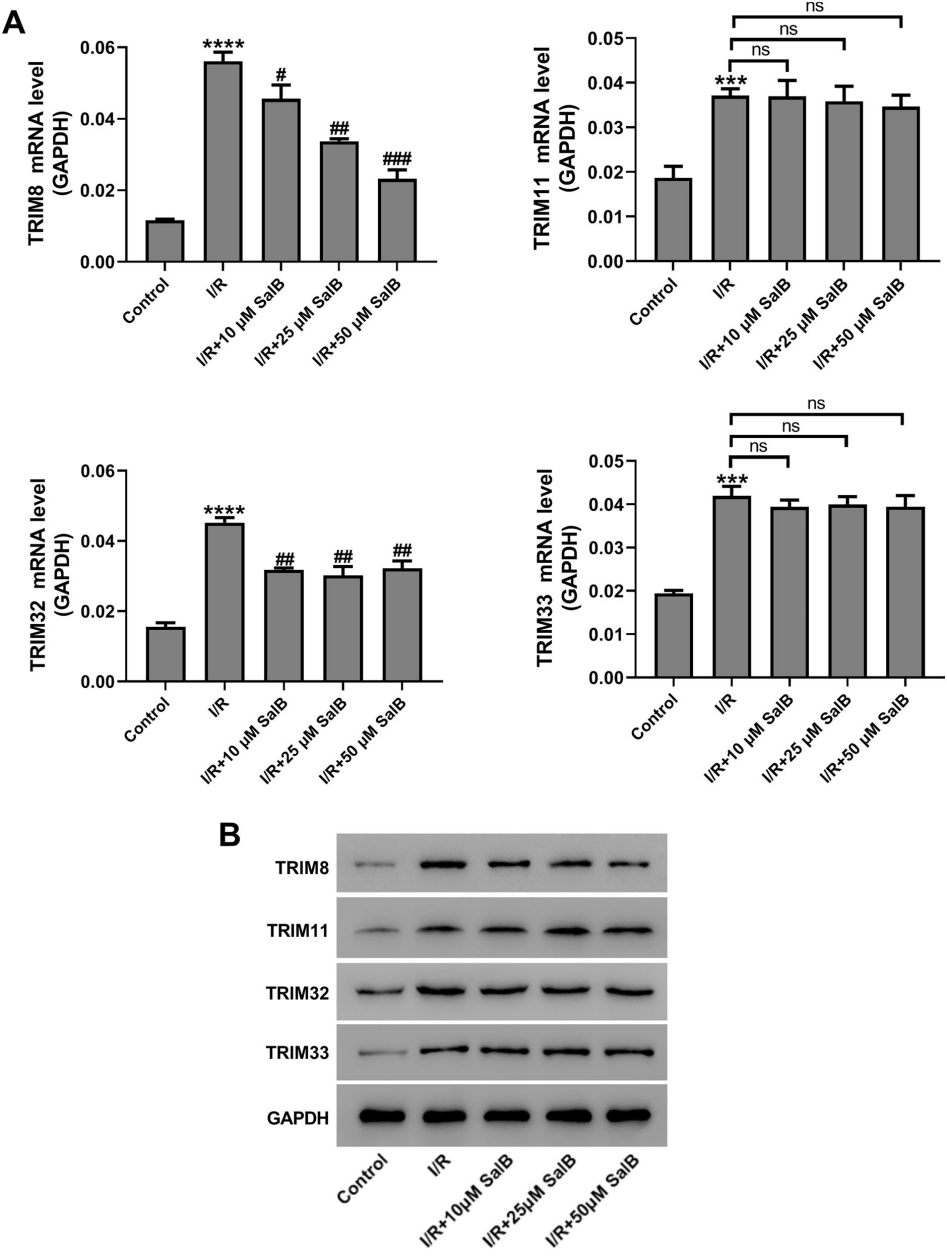
SalB inhibited ischemia/reperfusion (I/R)-induced up-regulation of TRIM8 expression. I/R-injured AC16 cells were treated with different concentrations of SalB. (A) The mRNA levels of TRIM8, TRIM11, TRIM32, and TRIM33 were detected by RT-qPCR. (B) The protein levels of TRIM8, TRIM11, TRIM32, and TRIM33 were detected by western blotting. ***p < 0.001 and ****p < 0.0001 vs. Control; ^#^*p* < 0.05, ^##^*p* < 0.01, and ^###^*p* < 0.001 vs. I/R. ns: not significant.

Next, we assessed the expression levels of four TRIM proteins, TRIM8, TRIM11, TRIM32, and TRIM33 by performing RT-qPCR and western blotting. Results showed that the expression levels of all four proteins were significantly increased in I/R-treated AC16 cells (*p* < 0.001, Figure 2). Subsequent SalB treatment didn’t affect the expression of TRIM11 and TRIM33, while it attenuated I/R-induced up-regulation of TRIM8 and TRIM32 (Figure 2). Interestingly, SalB at higher concentration showed more significant effect on TRIM8 expression-inhibition than that at lower concentration, while different concentrations of SalB had similar inhibitory effect on TRIM32 expression (Figure 2). Considering the sensitivity of TRIM8 to the concentration of SalB, it was selected for subsequent experiments.

### Knock-down of TRIM8 alleviated I/R-induced apoptosis and oxidative stress in AC16 cells

**Figure 3.**
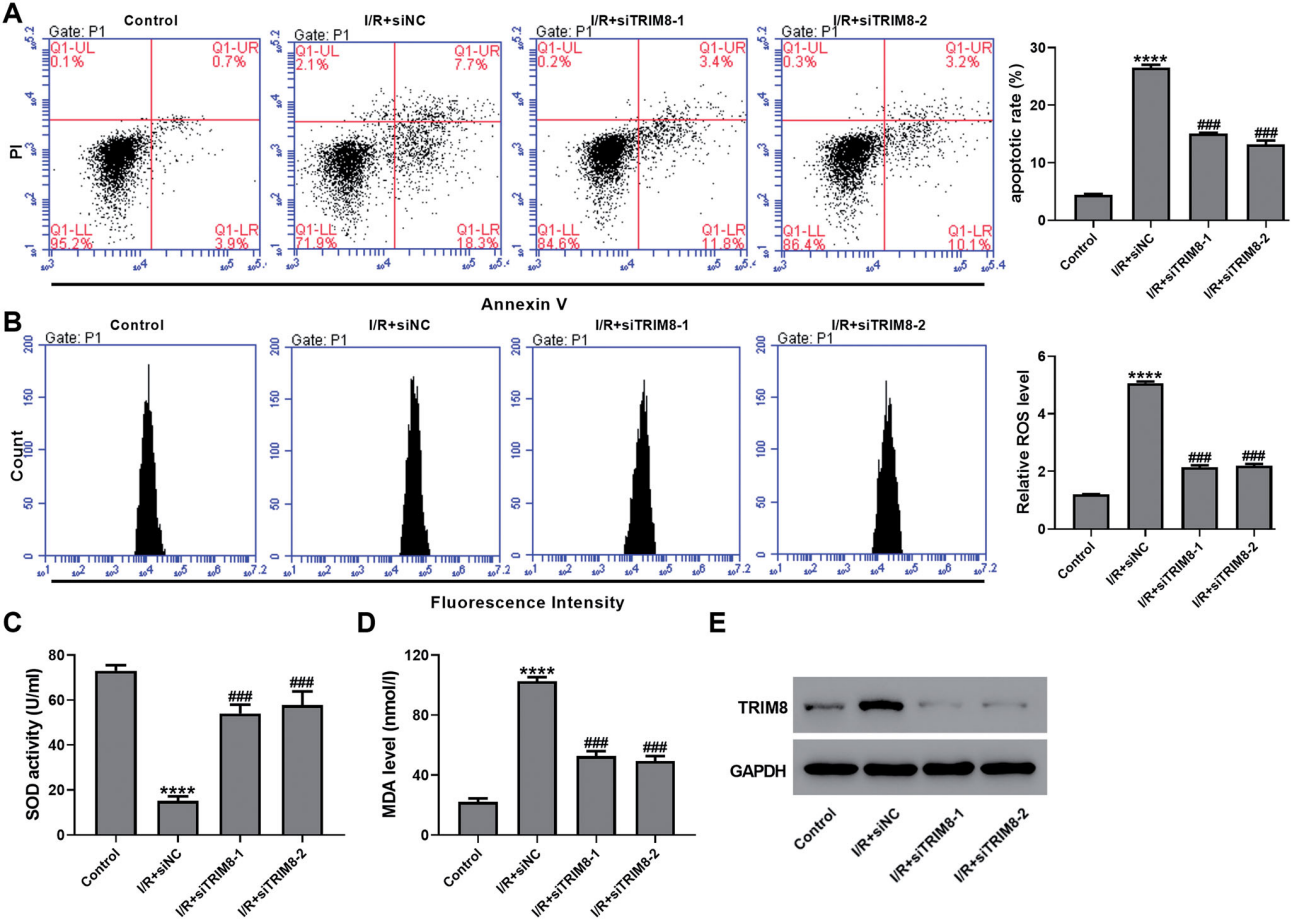
Knock-down of TRIM8 alleviated ischemia/reperfusion (I/R)-induced apoptosis and oxidative stress in AC16 cells. I/R-injured AC16 cells were transduced with lentivirus siTRIM8. (A) Apoptotic detection was performed using a flow cytometry. (B) ROS level was detected with DCFH-DA probe. (C) SOD activity was detected using xanthine oxidase method. (D) MDA level was detected using TBA method. (E) TRIM8 protein level was determined. ****p < 0.0001 vs. Control; ^###^*p* < 0.001 vs. I/R + siNC.

Lentivirus siTRIM8 and oeTRIM8 were designed to knock down and overexpress TRIM8 in AC16 cells, and their effectiveness was confirmed by RT-qPCR and western blotting (Figure S2). I/R-injured AC16 cells were transduced with control siRNA (siNC) or siTRIM8 (siTRIM8-1, siTRIM8-2), and cells that were cultured under normal conditions were used as control. Results showed that the apoptotic rate in I/R-injured AC16 cells were significantly increased (I/R + siNC, 26.6% ± 0.5% vs. Control, 4.4% ± 0.2%), while the up-regulated apoptotic rate were inhibited by TRIM8 knock-down (I/R + siTRIM8-1, 15.1% ± 0.2%; I/R + siTRIM8-2, 13.2% ± 0.7%) (Figure 3A). ROS level in AC16 cells with I/R treatment was increased to 4.2-fold of control cells, and dropped to 1.8-fold of control cells when siTRIM8-1 or siTRIM8-2 was applied. In addition, SOD activity was down-regulated by 79.3% and MDA level was enhanced by 3.6-fold in I/R-injured AC16 cells compared to control cells, while they were restored to normal level by TRIM8 knock-down (Figure 3C–D). Taken together, TRIM8 knock-down can inhibit I/R-induced apoptosis and oxidative stress in AC16 cells.

### SalB alleviated TRIM8 overexpression-induced apoptosis and oxidative stress in AC16 cells

**Figure 4.**
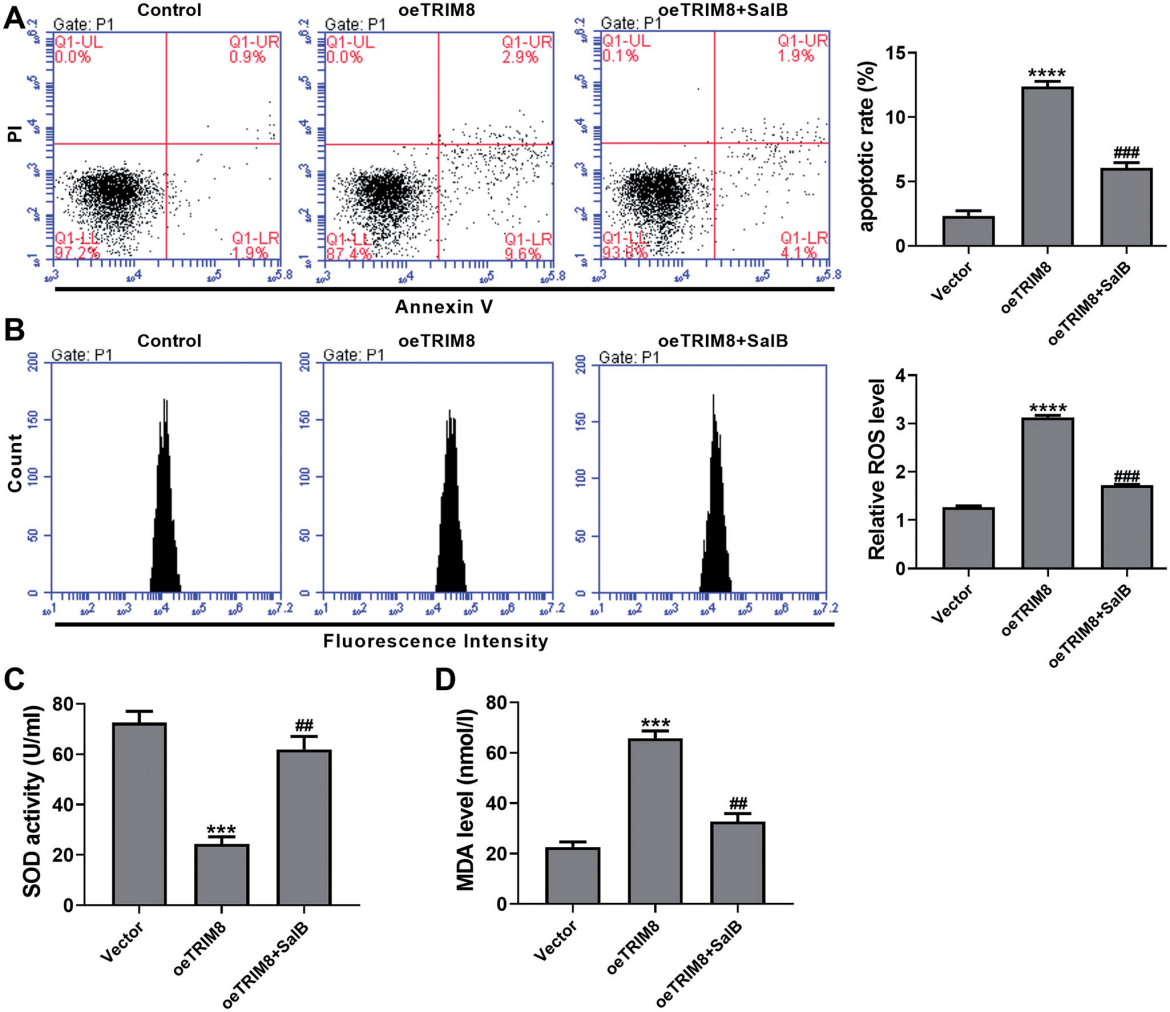
SalB alleviated TRIM8 overexpression-induced apoptosis and oxidative stress in AC16 cells. AC16 cells were transduced with lentivirus oeTRIM8, and the treated with 50 μM SalB. (A) Apoptotic detection was performed using a flow cytometry. (B) ROS level was detected with DCFH-DA probe. (C) SOD activity was detected using xanthine oxidase method. (D) MDA level was detected using TBA method. ***p < 0.001 and ****p < 0.0001 vs. vector; ##*p* < 0.01, and ###*p* < 0.001 vs. oeTRIM8.

Lentivirus oeTRIM8-transduced AC16 cells were treated with 50 μM SalB, and lentivirus vector-transduced AC16 cells were used as control. Results showed that TRIM8 overexpression markedly promoted apoptosis (oeTRIM8, 12.4 ± 0.4% vs. Control, 2.3% ± 0.4%) in AC16 cells, whereas the up-regulation of apoptotic rate was inhibited by subsequent SalB administration (oeTRIM8 + SalB, 6.1% ± 0.4%) (Figure 4A). ROS level in AC16 cells with TRIM8 overexpression was increased to 2.5-fold of control cells, and dropped to 1.4-fold of control cells when 50 µM SalB was applied (Figure 4B). In addition, TRIM8 overexpression significantly reduced SOD activity by 66.4% and increased MDA level by 1.9-fold compared to control cells, while they were restored to normal level by SalB administration (Figure 4C–D).

### TRIM8 negatively regulated GPX1 via ubiquitination

**Figure 5.**
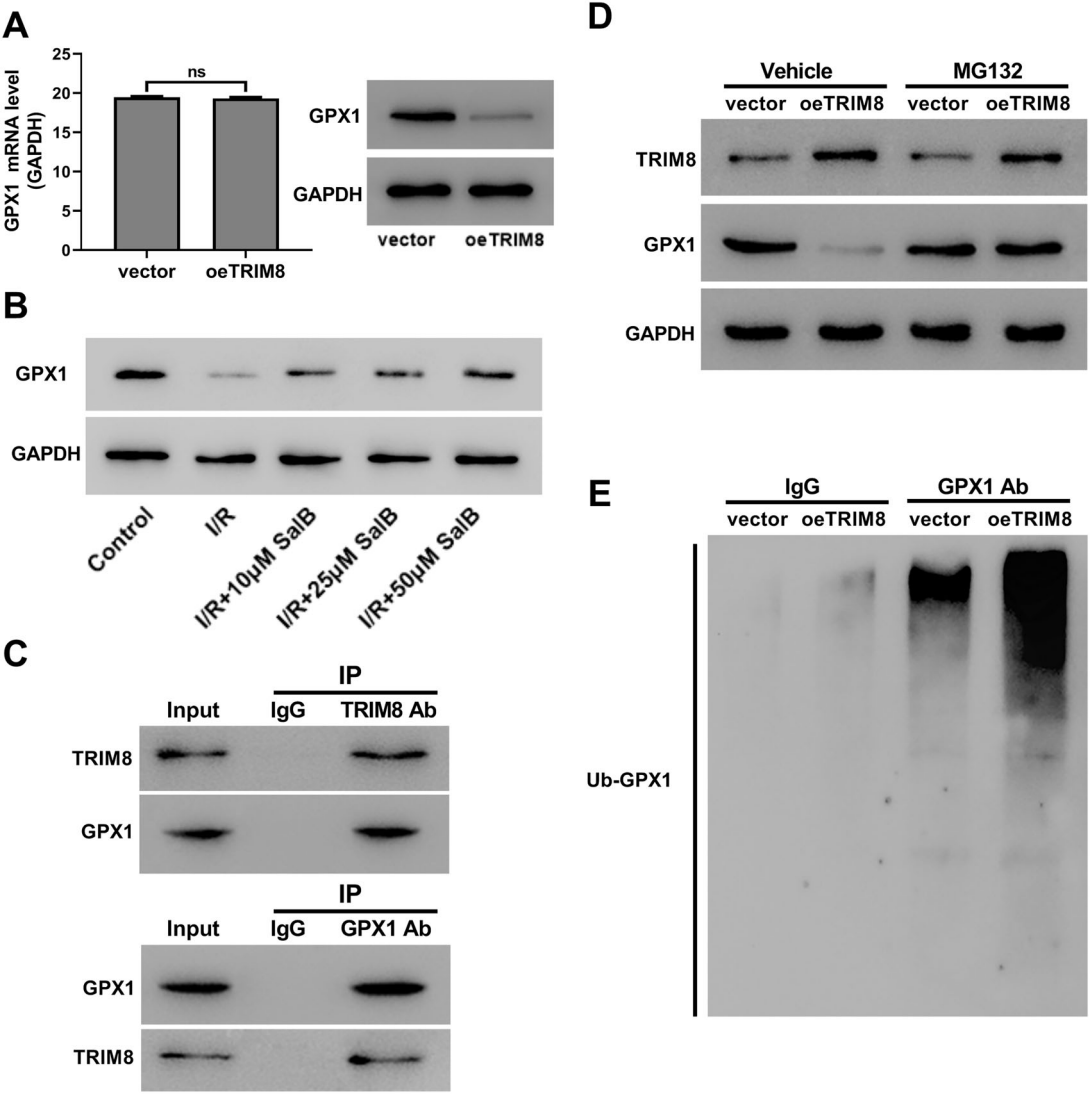
TRIM8 negatively regulated GPX1 via ubiquitination. (A) AC16 cells were treated with lentivirus oeTRIM8. TRIM expression level was assessed. (B) I/R-injured AC16 cells were treated with different concentrations of SalB. The protein levels of GPX1 were detected by western blotting. (C) Co-immunoprecipitation assay was performed to detect the interaction between TRIM8 and GPX1. (D) AC16 cells were treated with lentivirus oeTRIM8 and proteasome inhibitor MG132. TRIM8 and GPX1 protein levels were determined. (E) AC16 cells were treated with lentivirus oeTRIM8. The ubiquitination of GPX1 was detected by ubiquitination assay. ns: not significant.

We also detected the effect of TRIM8 on the expression of GPX1, an antioxidant enzyme from GPX family. Results showed that TRIM8 overexpression obviously reduced GPX1 protein but didn’t affect GPX1 mRNA (Figure 5A). SalB, which suppressed TRIM8 expression, induced GPX1 protein expression (Figure 5B). Co-immunoprecipitation assay showed that TRIM8 interacted with GPX1 (Figure 5C). In addition, proteasome inhibitor MG132 abolished TRIM8-mediated decrease of GPX1 protein (Figure 5D). Ubiquitination assays showed that TRIM8 overexpression promoted the ubiquitination of GPX1 (Figure 5E). Therefore, TRIM8 mediates the degradation of GPX1 via ubiquitination.

### GPX1 knock-down abolished the protective effects of SalB on I/R-injured cardiomyocytes

**Figure 6.**
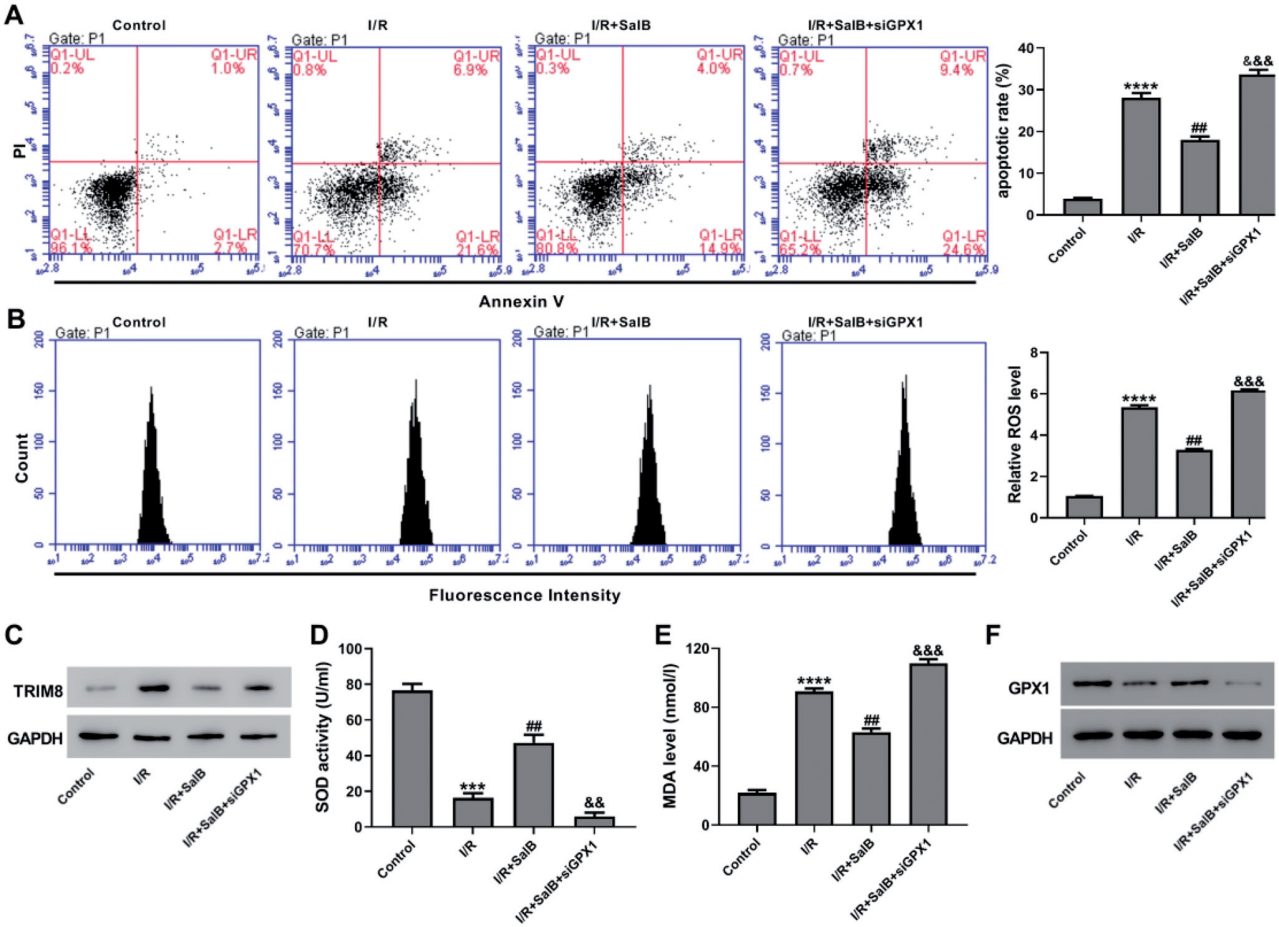
GPX1 knock-down abolished the protective effects of SalB on ischemia/reperfusion (I/R)-injured cardiomyocytes. I/R-injured AC16 cells were treated with SalB and lentivirus siGPX1. (A) Apoptotic detection was performed using a flow cytometry. (B) ROS level was detected with DCFH-DA probe. (C) TRIM8 protein level was measured. (D) SOD activity was detected using xanthine oxidase method. (E) MDA level was detected using TBA method. (F) GPX1 protein level was measured. ***p < 0.001 and ****p < 0.0001 vs. control; ^##^*p* < 0.01 vs. I/R; ^&&^*p* < 0.001 vs. I/R + SalB.

Lentivirus siGPX1 was designed to knock down the expression of GPX1 in AC16 cells, and its effectiveness was confirmed by RT-qPCR and western blotting (Figure S2). I/R-injured AC16 cells were treated with 50 μM SalB and then were transduced with siGPX1, while cells that were cultured under normal conditions were used as control. Flow cytometric analyses showed that SalB treatment to I/R-induced AC16 cells inhibited apoptosis by 36.0% (I/R + SalB, 18.0 ± 0.9% vs. I/R, 28.0% ± 1.2%) and ROS generation by 38%, compared to I/R-induced AC16 cells, while the effects of SalB on apoptosis and ROS generation were reversed by GPX1 knock-down (Figure 6A–B). TRIM8 protein expression was increased by I/R and decreased by SalB treatment (Figure 6C). In addition, SalB treatment increased SOD activity by 1.9-fold and reduced MDA level by 30.6% compared to I/R-induced AC16 cells, while he effects of SalB on SOD activity and MDA level were reversed by GPX1 knock-down (Figure 6D–E). Taken together, our data suggest that GPX1 knock-down abolished the protective effects of SalB on I/R-injured AC16 cells.

### SalB inhibited cardiomyocyte apoptosis and ROS generation by regulating TRIM8/GPX1 axis in vivo

**Figure 7.**
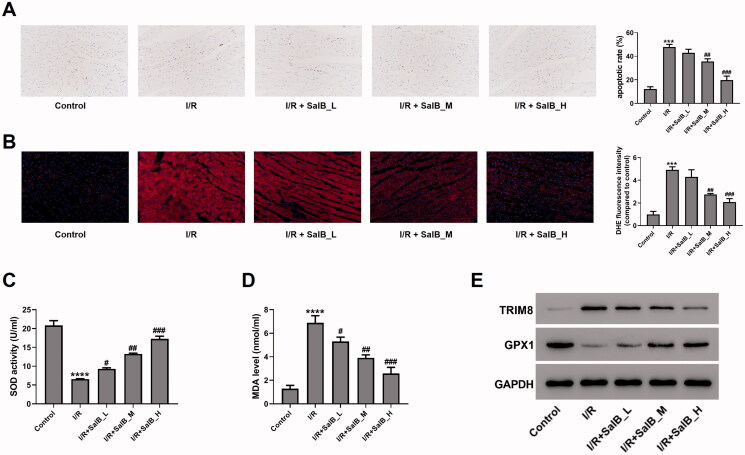
SalB inhibited cardiomyocyte apoptosis and ROS generation by regulating TRIM8/GPX1 axis *in vivo*. Ischemia/reperfusion (I/R)-injured rat model was treated with different concentrations of SalB (L: 20 mg/kg SalB; M: 40 mg/kg SalB; H: 60 mg/kg SalB). (A) Apoptotic detection was performed by TUNEL assay. (B) ROS level was detected with dihydroethidium staining. (C) SOD activity was detected using xanthine oxidase method. (D) MDA level was detected using TBA method. (E) TRIM8 and GPX1 protein levels were measured. ***p < 0.001 and ****p < 0.0001 vs. control; ^#^*p* < 0.05, ^##^*p* < 0.01, and ^###^*p* < 0.001 vs. I/R.

An *in vivo* rat model of I/R injury was established and subjected to SalB treatment, while control rats received sham operation and saline injection. TUNEL assay showed that I/R treatment promoted cardiomyocyte apoptosis by 3.0-fold over control (I/R, 47.9% ± 2.2%; Control, 12.1% ± 2.0%), while SalB treatment (SalB L, 20 mg/kg; SalB M, 40 mg/kg; SalB H, 60 mg/kg) attenuated I/R-induced apoptosis (I/R + SalB L, 43.0% ± 2.9%; I/R + SalB M, 35.6% ± 2.3%; I/R + SalB H, 19.8% ± 3.5%) (Figure 7A). Dihydroethidium staining showed that I/R treatment promoted ROS generation to 4.9-fold over Control, while the up-regulated ROS level was alleviated by SalB treatment to 4.3-, 2.7-, and 2.1-fod over Control at a dose of 20, 40, and 60 mg/kg, respectively (Figure 7B). In addition, I/R treatment reduced SOD activity and increased MDA level in cardiomyocytes, while the effects of I/R treatment on SOD and MDA were attenuated by I/R treatment (Figure 7C–D). Western blotting showed that I/R treatment increased TRIM8 protein and reduced GPX1 protein, while the effects of I/R treatment on these two proteins were attenuated by SalB treatment (Figure 7E). Taken together, our data suggested that SalB treatment suppressed I/R-induced cardiomyocyte apoptosis and ROS generation *in vivo*, and TRIM8/GPX1 axis might be involved in the regulatory process.

## Discussion

Recent studies have revealed that SalB can alleviate myocardial I/R injury (Qiao and Xu 2016; Liu et al. 2019). TRIM proteins have been recognised as important regulators in the pathological process of myocardial I/R injury (Jing et al. 2016; Yin et al. 2016). GPX1, an antioxidant enzyme, is responsible to scavenge the accumulated ROS in cells, thereby reducing the oxidative stress (Al Hadithy et al. 2010). Here, we first reported that SalB inhibits I/R-induced cardiomyocyte apoptosis and oxidative stress partly by TRIM8/GPX1 axis.

SalB is a phenolic compound which has antioxidant and free radical scavenging properties (Dong et al. 2010). Studies have found that SalB treatment attenuates cerebral I/R injury by regulating toll like receptor (TLR4)/MyD88 pathway (Wang et al. 2016), and alleviates renal I/R injury by regulating phosphatidylinositol 3-kinase (PI3K)/Akt pathway (Wang et al. 2013). Recently, several studies have revealed that SalB protects cardiomyocytes against I/R injury (Qiao and Xu 2016; Liu et al. 2019). Consistently with the previous studies, we found that SalB inhibited I/R-induced cardiomyocyte apoptosis and ROS generation both in cardiomyocyte cell line AC16 and rat model of myocardial I/R injury.

The molecular mechanisms how SalB protects I/R-injured cardiomyocytes are unclear (Qiao and Xu 2016; Liu et al. 2019). Previous studies have revealed that knock-down of TRIM8 can alleviate neuronal, hepatic, and cerebral I/R injury (Tao et al. 2019; Bai et al. 2020; Zhao et al. 2020). A recent study has reported that TRIM8 is up-regulated in rat cardiomyocyte cell line H9C2 exposed to I/R, and knock-down of TRIM8 inhibits cardiomyocyte apoptosis and ROS generation (Dang et al. 2020). Here, our study confirmed their findings in human cardiomyocyte cell line AC16. We found that I/R-induced cardiomyocytes had up-regulated TRIM8 expression, while the up-regulation of TRIM8 was inhibited by SalB treatment. TRIM8 knock-down repressed I/R-mediated cardiomyocytes apoptosis and oxidative stress. TRIM8 overexpression promoted cardiomyocytes apoptosis and oxidative stress, while the effects of TRIM8 were attenuated by SalB treatment. Moreover, SalB attenuated I/R treatment-increased TRIM8 protein expression in the rat model of myocardial I/R injury. Collectively, our data indicate that SalB inhibits I/R-induced cardiomyocyte apoptosis and oxidative stress by regulating TRIM8.

Existing studies have revealed that the antioxidant enzyme GPX1 was significantly reduced in I/R-injured cardiomyocytes (Thu et al. 2010). Our study showed that GPX1 overexpression alleviated I/R-induced apoptosis and oxidative stress in AC16 cells (Figure S3), suggesting the protective role of GPX1 in I/R-injured cardiomyocytes. Moreover, GPX1 knock-down abolished the protective effects of SalB on I/R-injured cardiomyocytes, indicating the involvement of GPX1 in the effects of SalB. A recent study has revealed that GPX1 expression in I/R-injured cardiomyocytes was down-regulated by TRIM33-mediated ubiquitination (Jian et al. 2016). TRIM8 is an E3 ubiquitin ligase which mediates protein degradation via ubiquitination (Ye et al. 2017). Here, ubiquitination-related assay revealed that TRIM8 mediated the degradation of GPX1 via ubiquitination. Thus, we speculate that in I/R-injured cardiomyocytes, GPX1 is negatively regulated by TRIM proteins-mediated protein degradation. Furthermore, our *in vivo* experiments revealed that I/R treatment promoted cardiomyocyte apoptosis and oxidative stress, increased TRIM8 protein, and reduced GPX1 protein, while the effects of I/R treatment were attenuated by SalB treatment. Collectively, we reported that TRIM8 functioned in the protective effects of SalB in I/R-induced cardiomyocyte apoptosis and oxidative stress by regulating GPX1 ubiquitination.

Some limitations exist for the current study. First, TRIM8 knock-down partially reverse myocardial I/R injury, which suggested other molecules may participate in myocardial I/R injury. We found that I/R treatment in cardiomyocytes up-regulated TRIM32, which was inhibited by subsequent SalB administration. Previous study has found that knock-down of TRIM32 can protect neurons against I/R injury (Wei et al. 2019). Abnormal expression of TRIM32 was observed in many heart diseases, such as heart failure and atrial fibrillation (Borlepawar et al. 2019). Therefore, we speculate that TRIM32 may also play a role in myocardial I/R injury, which deserves to be explored in the future. Second, although we found that TRIM8 expression was up-regulated in response to I/R injury *in vitro* and *in vivo*, it is unclear whether TRIM8 expression is altered in human myocardial infarction, which is to be clarified in the further investigations.

## Conclusions

We demonstrate that SalB inhibits I/R-induced cardiomyocyte apoptosis and oxidative stress *in vivo* and *in vitro.* The underling mechanism of SalB mediated cardioprotection may attribute to the down-regulation of TRIM8 and up-regulation of GPX1.Our study provides an in-depth insight into the regulatory mechanism of SalB on myocardial I/R injury, and suggested that down-regulation of TRIM8 expression may provide an efficient approach to ameliorate I/R-induced myocardial injury.

## Supplementary Material

Supplemental Figures
