# Supplementary material for: Salvianolic acid B inhibits myocardial I/R-induced ROS generation and cell apoptosis by regulating the TRIM8/GPX1 pathway
Source: Pharm Biol. 2022 Aug 14;60(1):1458–68. doi: 10.1080/13880209.2022.2096644 (PMC9380432; doi:10.1080/13880209.2022.2096644)
Supplement: Supplemental Figures [file IPHB_A_2096644_SM0792.docx]

**
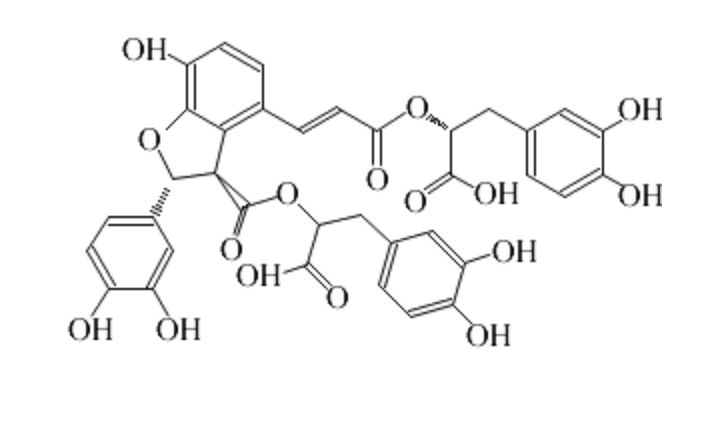
**

**Figure S1.** Structure of Salvianolic acid B.

**
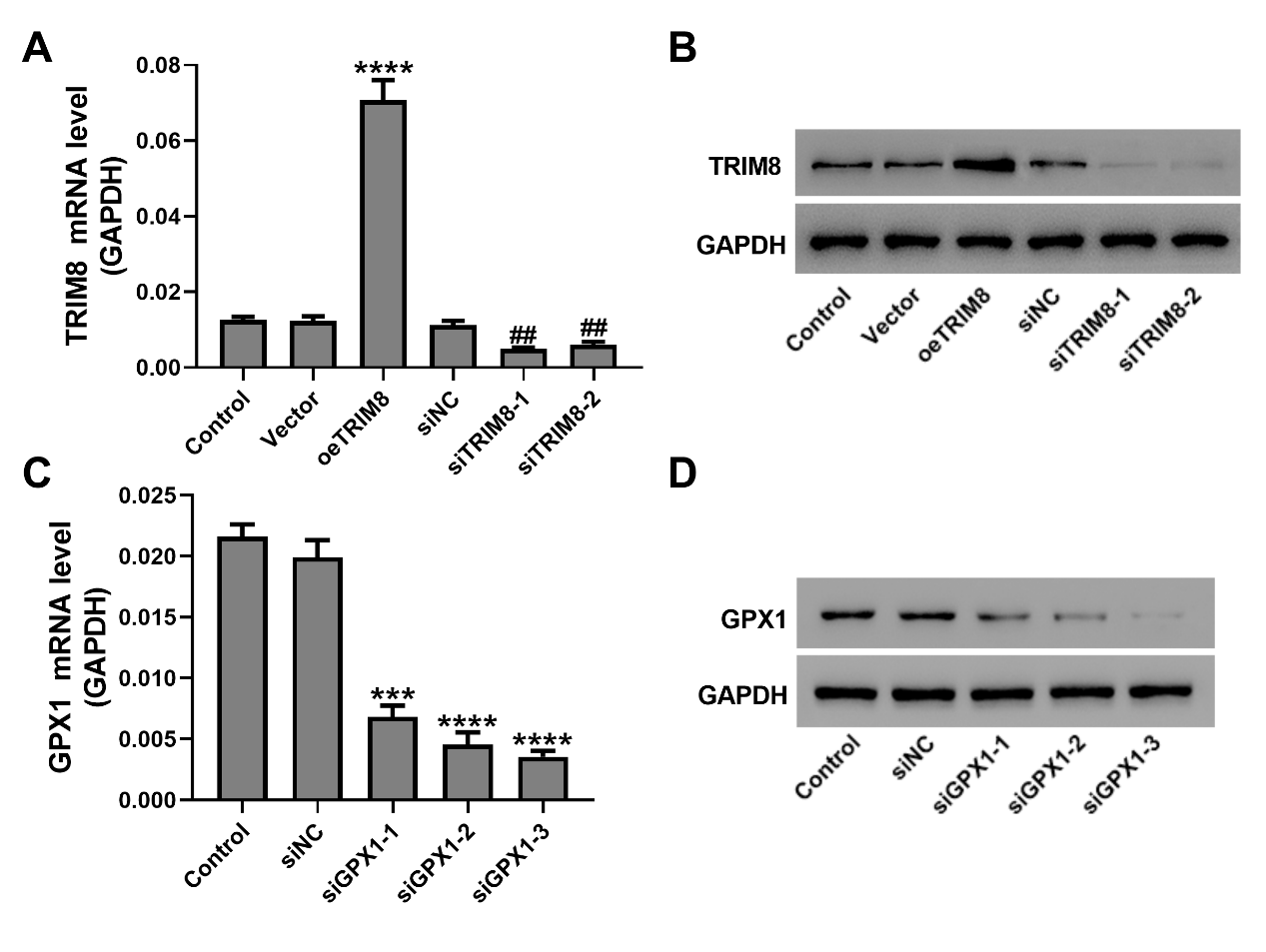
Figure S2. The expression levels of TRIM8 and GPX1 in AC16 cells.** (A-B) AC16 cells were transduced with lentivirus oeTRIM8 or siTRIM8. TRIM8 mRNA (A) and protein (B) levels were measured. (C-D) AC16 cells were transduced with lentivirus siGPX1. GPX1 mRNA (C) and protein (D) levels were measured. ***p<0.001 and ****p<0.0001 vs vector or siNC; ##p<0.01 vs siNC.


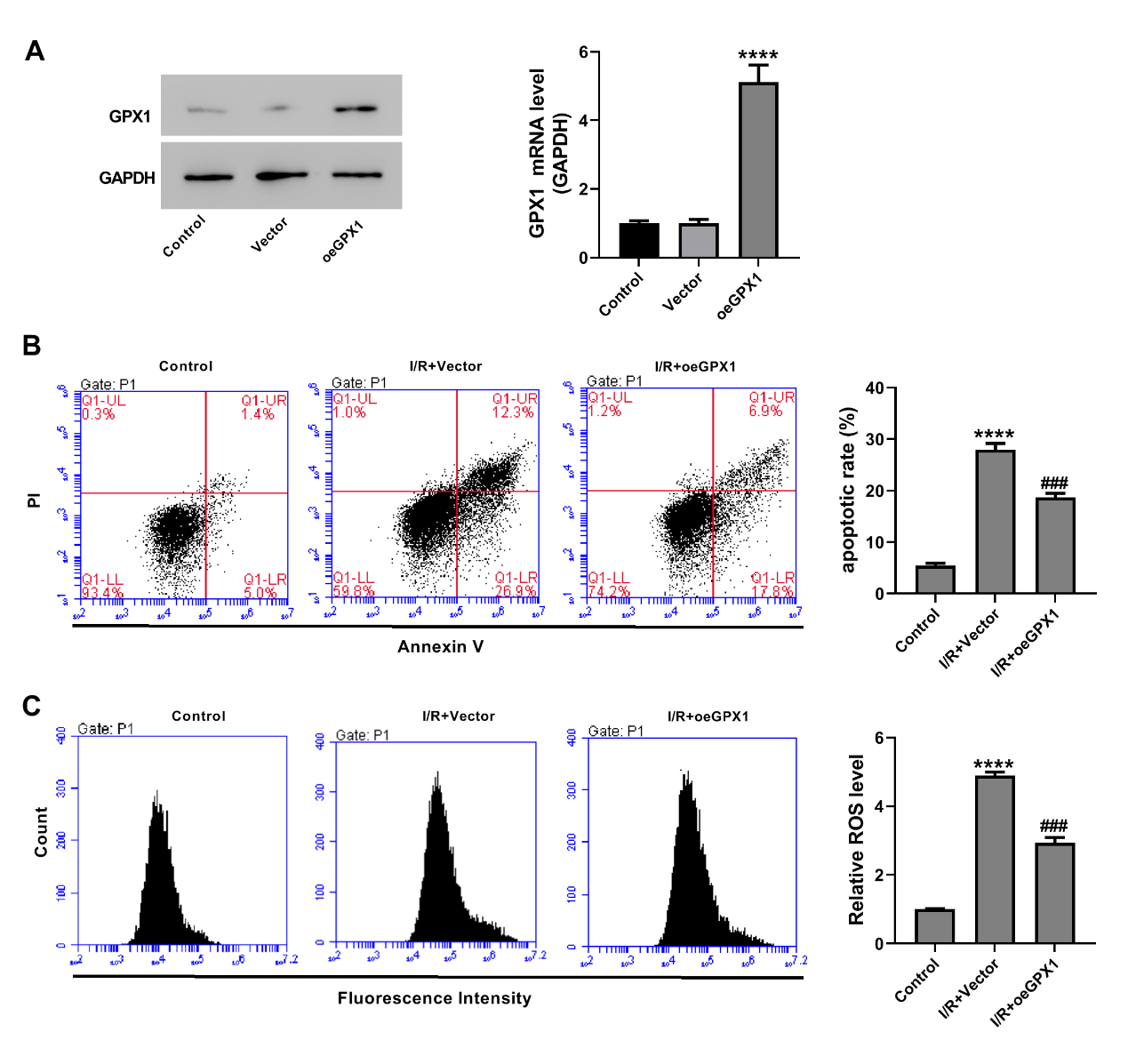


**Figure S3. GPX1 overexpression alleviated I/R-induced apoptosis and oxidative stress in AC16 cells**. (A) AC16 cells were transfected with plasmid expressing Vector or GPX1 (oeGPX1). GPX1 protein (left panel) and protein (left panel) levels were measured. ****p<0.0001 vs Control. (B-C) I/R-injured AC16 cells were overexpressed with GPX1 (oeGPX1). (B) Apoptotic detection was performed using a flow cytometry. (C) ROS level was detected with DCFH-DA probe. ****p<0.0001 vs Control; ###p<0.001 vs I/R+siNC.
